# Effect of Fresh Frozen Plasma Infusion on Hospital Length of Stay for Patients With Hereditary Angioedema

**DOI:** 10.36469/001c.141171

**Published:** 2025-07-09

**Authors:** Subhan Khalid, Alan T. Hitch

**Affiliations:** 1 Harrisburg University of Science and Technology

**Keywords:** Bayesian additive regression trees, hereditary angioedema, epidemiology, fresh frozen plasma infusion, hospital length of stay, treatment effect

## Abstract

**Background:** Patients with hereditary angioedema treated with fresh frozen plasma (FFP) infusion face complications and risk of side effects.

**Objective:** To study the effect of FFP infusion on hospital length of stay for patients with hereditary angioedema.

**Methods:** Data from the 2021 Nationwide Inpatient Sample were used to identify hospitalized patients with hereditary angioedema. Patient demographics, comorbidities, and severity measures were analyzed, and a Bayesian additive regression tree model was used to assess factors contributing to length of stay.

**Results:** FFP infusion was found to be associated with increased length of stay for patients with risk factors such as respiratory, cardiovascular disease, or urticaria.

**Conclusions:** Caution is recommended when planning to use FFP, to ensure that underlying patient conditions and risk factors are thoroughly understood. The findings emphasize the need for personalized treatment plans based on individual risk factors, with a recommendation for prioritizing C1-inhibitor therapy over FFP.

## BACKGROUND

Hereditary angioedema (HA) is a rare genetic condition characterized by recurrent swelling episodes in various parts of the body, including the skin, upper airway tracts, and respiratory system.^1^ The underlying cause of HA is most often a deficiency or dysfunction in the C1 esterase inhibitor (C1-INH) protein, which helps regulate the complement system. The condition is caused by mutations in the SERPING1 gene, which encodes the C1-INH protein, and follows an autosomal dominant inheritance pattern that can manifest at any age. Attacks can occur unpredictably, triggered by stress, infections, or trauma, which may worsen symptoms and lead to hospitalization.^2,3^

HA is classified into 3 types: type I involves low levels of C1-INH, type II involves dysfunctional C1-INH, and type III involves normal C1-INH levels but mutations in the F12 gene that affect factor XII.^4^ Type I is the most common, accounting for 85% of cases, type II accounts for nearly 15%, and type III is very rare.^4^ Blood tests are conducted to assess C1-INH levels, including checking for low C4 levels, which drop during attacks. For a definitive diagnosis, molecular testing is used to detect SERPING1 mutations. Due to its variable presentation, HA is often underdiagnosed or misdiagnosed, leading to delayed treatment.^5^

Treatment for HA focuses on reducing symptoms, such as managing acute attacks, preventing future episodes, and improving long-term quality of life. The primary approach to treating acute attacks involves C1-INH replacement therapy administered through the bloodstream to replenish the deficient or malfunctioning C1-INH levels. Other treatments include medications such as bradykinin receptor antagonists, which treat acute attacks, and icatibant, which blocks bradykinin’s effects, and kallikrein inhibitors, which prevent bradykinin production. If these options are not available, fresh frozen plasma (FFP) has been used to treat acute attacks since it contains functional C1-INH and helps regulate bradykinin production, helping prevent^6^ or provide relief from an HA attack.^7^ FFP is not the first choice of treatment since it carries the risk of transmission of infections and is not as readily available as other treatments.^8,9^

For long-term management, patients who experience frequent and/or severe attacks use prophylactic treatments to reduce the frequency and intensity of episodes. These treatments include C1-INH infusions or androgens (such as danazol), which stimulate the liver to produce more C1-INH. However, androgens carry side effects, including liver damage and hormonal changes.^9^ Gene therapy and RNA-based treatments are other approaches being researched as potential long-term solutions for HA with fewer side effects.^9^

C1-INH concentrates remain the most widely used treatment in Canada and the United States.^10^ However, there is significant variation in treatment times due to issues such as insurance approval and availability. Delays in receiving treatment result in longer episodes and more frequent visits to the emergency room (ER). Real-world studies, which generate clinical evidence outside controlled clinical trials, have evaluated the effectiveness of newer therapies such as icatibant and ecallantide.^11^ Frank et al found that these newer treatments significantly reduced the time required to resolve HA symptoms and decreased hospital admissions. Patients who used these therapies reported better symptom control and improved quality of life compared with those relying on older treatments such as FFP infusions.^11^

The HA literature emphasizes the importance of early diagnosis and timely treatment access to improve quality of life and reduce HA’s disease burden.^8,12^ Self-treatment, new biological therapies, and prophylactic approaches are increasingly key to effective management. However, access to these therapies must be improved to ensure better outcomes for all patients with HA, especially within the United States.

Studying the hospital length of stay (LOS) for patients with HA is crucial to understanding how to better manage symptoms, prevent adverse events, and lower costs. Extended hospital stays indicate delayed diagnosis, causing a longer time to improve patient condition. Longer LOS increases the risk of hospital-acquired infections and complications, while a premature discharge may lead to readmissions and adverse health effects.^13^ Modeling LOS can help identify factors that contribute to longer patient stays and develop treatments that can be used to minimize cost^14^ and lead to faster discharge.^15^ Variations in LOS may due to differences in hospitals, specialized care, and geographic factors.^16^

FFP has been a controversial treatment for HA due to concerns of potential exacerbation of symptoms. FFP contains proteins that can activate the complement system, which can potentially worsen an HA attack,^17^ cause an allergic reaction, or lead to transfusion-related complications.^18–20^ While it has been reported to be effective for some patients, others report no improvement.^21^ With the rollout of various new HA medications, such as C1-INH concentrates, the role of FFP in managing HA has become increasingly limited. FFP infusion is not recommended by the US Hereditary Angioedema Association medical advisory board as a safe treatment option.^22^ However, FFP continues to be administered in clinical settings, due to a lack of awareness of improved treatments or guidance related to its complications, prompting ongoing debate about its safety and effectiveness.

Early administration showed FFP as a safe and effective treatment,^23^ demonstrating reduced LOS.^24^ Some studies suggest that it does not exacerbate patient conditions,^25,26^ while others found no differences in LOS for critically ill patients receiving FFP.^27–29^

This analysis examines the treatment effect of FFP infusion on hospital LOS for HA patients, adjusting for demographic and clinical variables. With the gaps in current literature, further research is needed to fully understand its effects. The objective of this study is to examine whether FFP exacerbates patient conditions and increases LOS. Each HA patient is unique, and FFP might be effective for some, ineffective or dangerous for others. This study will attempt to exhibit scenarios where FFP is associated with longer LOS and should not be administered.

## METHODS

The Nationwide Inpatient Database (NIS) for 2021 was used for analysis. The NIS is the largest publicly available inpatient healthcare database in the United States. It contains records of inpatient utilization, access, cost, quality, and outcomes from approximately 7 million hospital stays yearly.^30–33^

*International Classification of Disease, Tenth Revision* (ICD-10) diagnosis code D84.1, defined as defects in the complement system, was used to identify patients who experienced an HA attack and were admitted to a hospital. This code is most commonly used for reimbursement purposes and enables us to capture HA patients who were diagnosed with HA and not with some other condition.^34^ A total of 441 patients were assigned to the HA cohort, where 70% were female, 58% were White, and 39% were in the 40- to 64-year age category (**Supplementary Table S1**). Private insurance (40%) was most prominently used to cover healthcare expenses. Most patients(80%) lived in urban areas, 37% were in the South, and 23% were in the West. Patient numbers were similar across income percentiles, with slightly higher proportions in the 51st to 75th percentiles.

Comorbidities can complicate patient conditions and extend hospital LOS. For HA patients, 48% had hypertension; obesity and chronic obstructive pulmonary disease (COPD) were each present in over 25%; and 20% had diabetes (**Supplementary Table S2**). A modified version of the Charlson Comorbidity Index (CCI)^35^ was used to quantify patient comorbidity burden. The original CCI includes conditions that were infrequently present or not consistently coded in our population of HA patients within the NIS database. Therefore, we developed a simplified CCI variant based on available baseline comorbidities. Alcohol use, autoimmune disease, depression, hypertension, thyroid disorder, peripheral vascular disease, drug abuse, and obesity were assigned a weight of 1, while chronic obstructive pulmonary disease (COPD) and diabetes were assigned a weight of 2. Diabetes was assigned a higher score as per the CCI or ICD-10–based mappings from Quan,^36^ while hospitalized HA patients usually need respiratory treatment due to swelling of passageways, presenting a higher burden for them. The CCI score ranged from 0 to 8 (**Supplementary Table S2**). Lower scores were more common, and only 8 patients had a score of ≥5.

**Figure 1.**
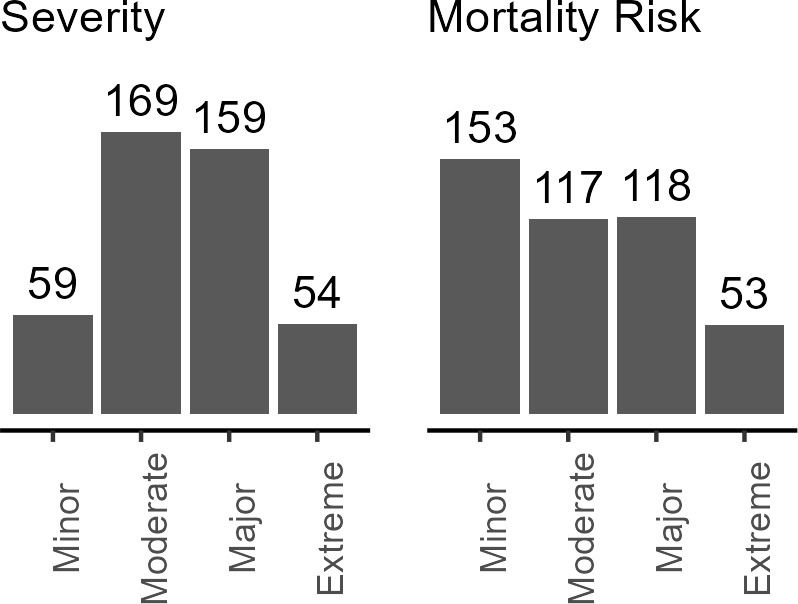
Severity of Patient Condition and Mortality Risk at Admission in the HA Cohort (N = 441) Abbreviation: HA, hereditary angioedema.

Patient severity and mortality risk at admission were derived in the NIS database using a classification system based on diagnosis, comorbidities, procedures, age, lab results and other patient factors, developed by 3M Health.^30^ Most patients were in the moderate and major categories for both measures (**Figure 1**). Fewer patients were classified at the extreme ends—either minor or extreme severity. Minor and moderate risk categories were used to create low risk, while major and extreme risk categories were used to create high-risk categories.

**Figure 2.**
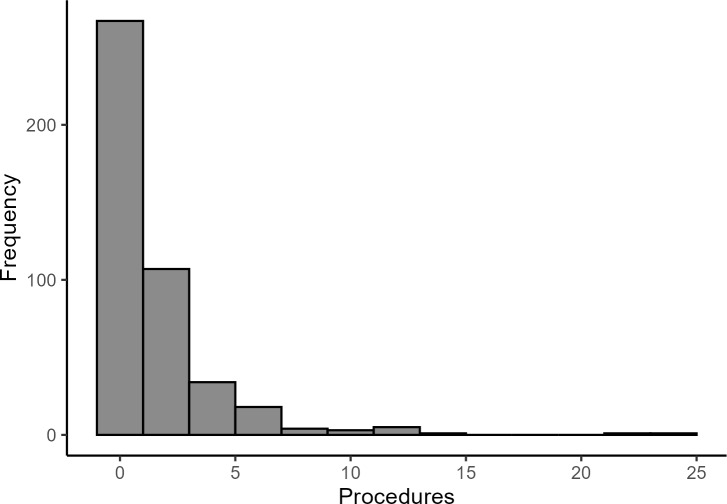
Distribution of Total Procedures Administered in the HA Cohort (N=441) Abbreviation: HA, hereditary angioedema.

Most HA patients were first seen in the ER rather than directly hospitalized (**Supplementary Table S3**). The ER-admitted patients were likely to present with more severe HA attacks requiring immediate attention. A large proportion of patients had no procedures during their hospital stay, while most underwent fewer than 5 (**Figure 2**). ICD-10 procedure codes 30233K1, 30233L1, and XW13325 were used to identify patients who received an FFP infusion, with 29 (6%) HA patients receiving it (**Supplementary Table S3**). Most received only 1 infusion, and only 4 received 2. Nearly all treated patients (28) were admitted through the ER.

During hospitalization, the most frequently diagnosed risk factor (**Supplementary Table S2**) was respiratory disease (n=97), followed by diabetes (n=85). Other common risk factors included cardiovascular disease (CVD), systemic lupus erythematosus (SLE),^37^ renal conditions (n=51), and urticaria.^38^ Less common risk factors included COPD, seizures, and sepsis. The ICD-10 diagnosis codes for risk factors are shown in **Supplementary Table S4**.

**Figure 3.**
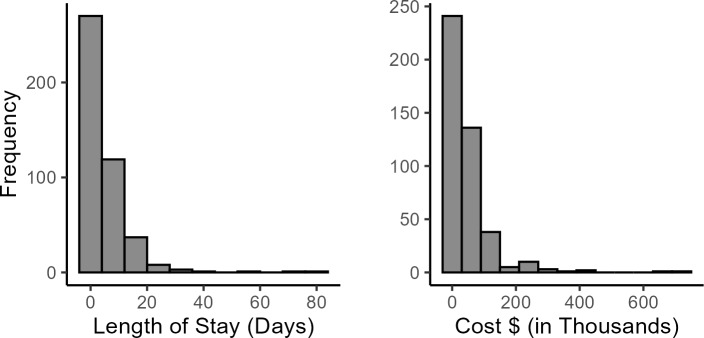
Distribution for Length of Stay (Days) and Cost ($) in the HA Cohort (N = 441) Abbreviation: HA, hereditary angioedema.

The cost of health care showed a right-skewed distribution, mostly clustered between $0 to $200 000 (**Figure 3**). The distribution for the outcome variable, hospital LOS, was also right-skewed, with most observations clustered around 0 to 20 days.

A Bayesian additive regression tree (BART) model was used to analyze the LOS. BART offers the flexibility of an ensemble decision tree model and the ability to set priors based on existing knowledge. It is particularly useful for modeling non-linear relationships and irregular patterns,^39–41^ as it does not make parametric assumptions about data distribution.^42^ This is beneficial when modeling right-skewed outcomes like LOS. As an ensemble model, BART performs well without requiring large datasets.^43^ Given the low number of FFP-treated patients, this modeling approach ensured adequate representation across trees for inference.

Computationally, BART begins by modeling the response variable as the sum of m regression trees.^44^ Each tree contributes a portion of the predicted value, and the sum of these contributions forms the overall model prediction. Initially, the trees are constructed with simple structures, then a Bayesian back-fitting Markov chain Monte Carlo algorithm is used to update each tree iteratively and its terminal node parameters, holding the others fixed.

The covariates included for the LOS model are listed in **Equation 1**. All analyses were conducted in R version 4.4.1 using the packages BART^45^ and tidybayes^46^ Interaction terms between the CCI and cost, CCI and severity, and cost and mortality risk were included in the model to better explain variations in hospital LOS among patients with longer time to discharge. Including interaction terms is important, as patient LOS can depend on a combination of variables and complex interdependencies, the omission of which can lead to model misspecification.^47,48^ The use of the interaction terms helps in accurately modeling hospital LOS, ensuring accurate inferences of treatment effect by subgroups.

(1)LOS=f(Age, Gender, Race, Income, Region, Payer, Severity,Mortality Risk, Cost, CCI, ER, FFP, Total Procedures,COPD, Sepsis, Renal Status, Systemic Lupus Erythematosus, Seizures,Urticaria, Mortality Risk × Cost, CCI × Cost, CCI × Severity)

where LOS is the outcome variable and *f(X)* represents the BART

model, which is a sum of trees:

$$f(X)=\sum_{k=1}^{K}T_{k}(X)$$

where *T^k^(X)*. These are individual regression trees. The interaction terms help the model to capture the combinative effects of variables.

The function *f(X)* represents an unknown, complex, and nonlinear relationship between the predictors and outcome. Fifty random trees were generated for each of the 40 000 Markov chain Monte Carlo (MCMC) iterations. The number of trees used (50) is recommended to model complex relationships and avoid overfitting.^49^ This is particularly helpful, as several covariates had to be included to model outlier LOS values. For the MCMC iterations, 10 000 were used as burn-in, while 30 000 were used as sampling. The burn-in phase consists of initial iterations that allow convergence to the target posterior distribution. Posterior samples are drawn for inference in the sampling phase, representing the true posterior distribution of the model parameters.

Priors regulate the number of trees and the depth of each tree, promoting a balance between complexity and generalization.^50^ Since the LOS for FFP infusion has not been studied in the literature extensively, we have used noninformative priors, so as to not place any restriction on the outcome, and let the data guide the posterior distribution. The following priors were used: learning the tree structure (number of trees, terminal nodes) was based on a Poisson distribution (λ = 2). The split decision for the tree was made based on a uniform distribution, meaning if there are *p* predictor variables, each variable could be chosen with probability 1/*p*. Leaf values were based on a normal prior, with mean 0 and variance τ² (N(0, τ²)). A smaller τ² leads to more regularization, preventing extreme predictions. The error term had a normal distribution with mean 0 and variance σ² (N(0, σ²)).

BART is a powerful tool for prediction, but improper tuning of hyperparameters can lead to overfitting or underfitting,^51^ it can be computationally expensive^52^ and it may experience performance degradation when handling a high number of covariates.^53^

## RESULTS

This study showed that FFP infusion was associated with increased LOS for patients with respiratory or CVD, with urticaria, or experiencing severe HA attacks.

**Figure 4.**
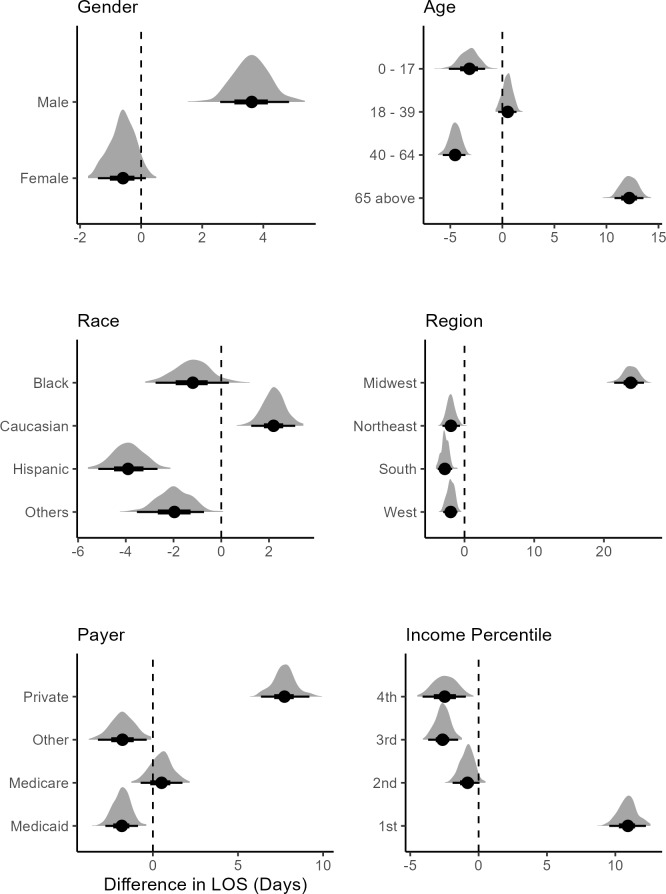
Posterior Difference Distributions between Treated and Untreated Patients Conditional on Demographics Note: The bold dot is the mean posterior difference; the dark black lines show the 95% credible interval. The vertical line shows which variable is significant.

Posterior predicted difference distributions were used to compare the LOS between treated and untreated patients.^54^ This is similar to computing contrast for comparison in an ANOVA setting. The posterior difference calculates the difference between the posterior distribution for LOS between 2 treated subgroups to make inferences about the effect of treatment. Female sex was associated with a lower mean difference in LOS, referred to hereafter as mean LOS, showing treated female patients being discharged sooner from the hospital than males (**Figure 4**). Male treated patients were associated with a higher mean LOS, showing longer times in the hospital for males receiving FFP infusion. For the ≥65 age group, treated patients were associated with longer stays in the hospital, while the younger age groups were associated with lower mean LOS. For White patients, treatment was associated with a higher mean LOS. Black, Hispanic, and other races were associated with lower mean LOS between treated and nontreated groups. In the Northeast, Midwest, and West, treatment was associated with lower mean LOS, indicating a treatment effect. In the Midwest, the treated patients were associated with substantially higher mean LOS.

**Figure 5.**
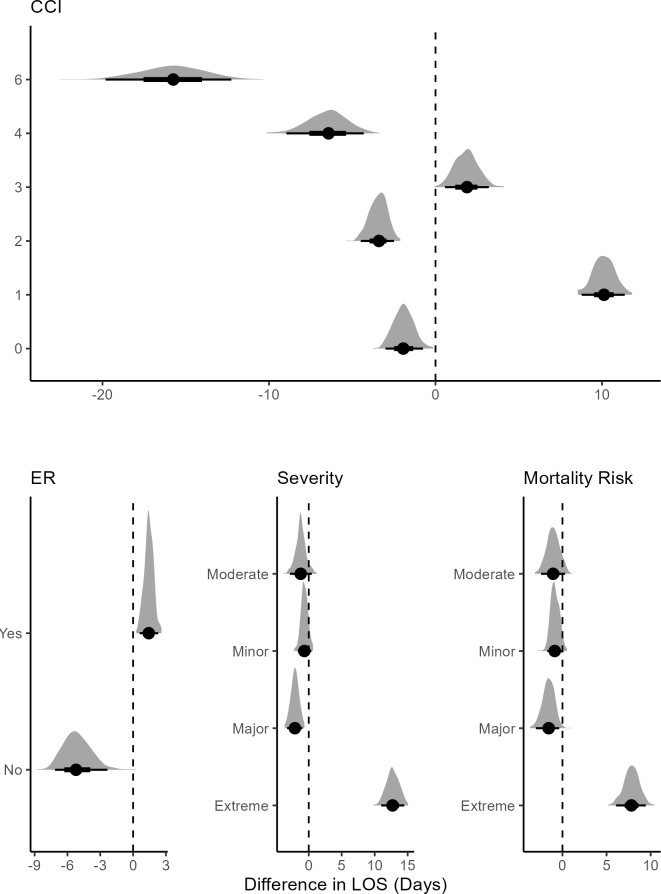
Posterior Difference Distributions Between Treated and Untreated Patients Conditional on CCI, ER Visit, Severity, and Mortality Risk Abbreviations: CCI, Charlson Comorbidity Index; ER, emergency room; LOS, length of stay. The bold dot shows the mean for the distribution; the dark black lines show the 95% credible interval. The vertical line shows which variable is significant using the credible interval. Note: There were no patients treated with scores of 5, 7, and 8.

Not being initially admitted to the ER was associated with lower mean LOS, while being admitted to the ER first was associated with higher (**Figure 5**). Treated and nontreated groups showed little discernible difference for minor or moderate severity. In patients with major severity, FFP treatment was associated with lower mean LOS than the nontreated group. Among patients with extreme severity, the treatment was associated with a high mean LOS. A similar pattern emerged for mortality risk.

At CCI scores 1 and 3, treatment was associated with an increase in mean LOS compared with the untreated group, with level 1 showing a notable increase (**Figure 5**). At CCI levels 4 and 6, the treatment effect was larger, with patients being associated with lower mean LOS. This suggests that FFP treatment may have a strong effect on LOS for moderate comorbid patients. None of the treated patients had CCI scores of 5, 7, and 8. For the last 2 levels, FFP might not have been considered, as it could potentially cause complications.

**Figure 6.**
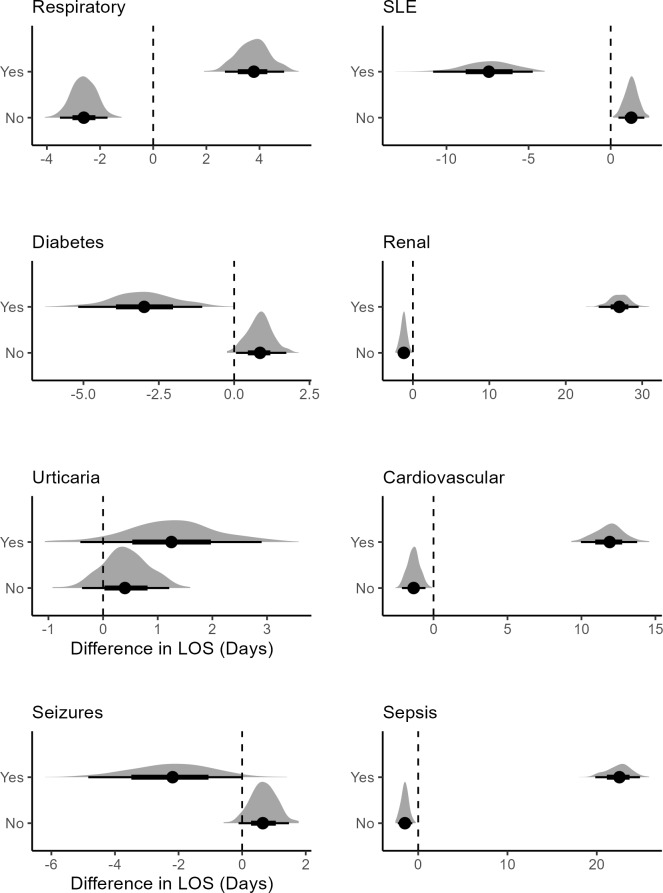
Posterior Difference Distributions Between Treated and Untreated Patients Abbreviation: LOS, length of stay; SLE, systemic lupus erythematosus.

For patients with SLE and diabetes, treatment was associated with lower mean LOS (**Figure 6**). While respiratory, urticaria, renal conditions, and cardiovascular patients that were treated were associated with higher mean LOS, which shows that FFP infusion should not be considered for HA patients developing these risk factors.

The ranking of covariates shows the frequency of being included in a decision tree as a splitting node divided by the total number of splitting rules appearing in the model (**Supplementary Figure S1**). Plot A showed respiratory disease, CCI × cost, income, and renal conditions appearing most frequently, indicating that these factors are important for predicting LOS. Other predictors (sepsis, cardiovascular, SLE) appeared regularly, suggesting they contribute meaningfully but to a lesser degree. Plot B showed how often a covariate was included in the same tree where the FFP treatment variable was included. Here, total procedures, income, region and respiratory disease appear most frequently. Urticaria, renal conditions, and sepsis also ranked highly. However, variables that are lower in order did not appear in trees with FFP but could still be important in the decision process for FFP infusion. The visualization of covariates can be used by other researchers to reproduce the results of our study.

## DISCUSSION

The use of FFP infusion in HA patients appeared to be primarily for patients admitted through the ER, with most patients receiving their first infusion on the same day as admission. This suggests that FFP was used as a first-line therapy to manage acute HA attacks. Based on the results, caution is recommended when using FFP and evaluating patients on a case-by-case basis prior to infusion. As discussed below, FFP use for patients with certain risk factors can exacerbate patient conditions rather than alleviate their symptoms. We also find a decline in the total number of procedures for HA patients after being administered 5 procedures. This suggests that procedural intervention for HA patients was less utilized; rather, some physicians relied on medication for HA management.^55,56^ Based on this study, we agree with this approach and suggest that therapies such as C1-INH be used to manage HA attacks, and FFP only used as a last resort.^57,58^ While FFP can be an effective intervention for acute HA attacks for certain patients, its use should be carefully evaluated based on individual patient risk factors, with priority given to therapies such as C1-INH to ensure optimal patient outcomes.

HA patients often face respiratory issues due to throat swelling caused by HA, which can affect the respiratory tract, the larynx, and the throat.^59^ Respiratory involvement in HA or laryngeal attacks can obstruct the airway and can be fatal, causing difficulty breathing and leading to asphyxiation or fatality.^60^ In our study, FFP in respiratory patients was associated with higher mean LOS, suggesting the infusion prolongs the stay in hospital. Based on the literature, we would suggest FFP be used as a temporary alternative or for emergency use where fatality could occur.^61,62^ If available, C1-INH concentrates should be the first-line therapy.^63–65^ Given the risks associated with respiratory involvement in HA, FFP should be reserved for emergencies where no alternative is available, with C1-INH concentrates prioritized as the first-line treatment for HA management.

Renal disease in HA patients may arise as a result of autoimmune conditions or as a result of frequent HA episodes that cause damage to renal tissues.^66^ HA patients may also experience urticaria (hives) due to the underlying dysregulation of the kallikrein-kinin system, leading to excessive bradykinin production.^67^ HA patients treated with medications like C1-INH, exposed to environmental allergens, or experiencing stress might develop urticaria as a side effect.^68^ It may also exist in HA patients with autoimmune conditions or other hypersensitivity reactions.^69^ A higher mean LOS was associated with renal and urticaria patients administered FFP. There is some guidance on FFP use for renal conditions,^70,71^ while there is none for urticaria. Based on our study, we do not recommend its usage for renal or urticaria patients experiencing HA attacks, as it could further aggravate patients’ conditions and lead to complications. Given the potential risks, its use may prolong hospitalization and exacerbate their condition, highlighting the need for alternative treatment approaches. Since the renal category for treated patients included as few as 2 patients, the findings should therefore be interpreted with caution and not considered as confirmatory.

CVD and hypertension are important considerations in HA patients, as both conditions may increase complications and influence treatment outcomes. HA patients could face an increase in the chances of hypertension, potentially leading to CVD.^72^ FFP usage was associated with higher mean LOS for CVD patients. FFP contains various plasma proteins and components that could influence coagulation, and in patients with CVD, the administration of FFP might exacerbate these issues.^73^ Given the complications that could arise for patients with hypertension or CVD, we do not recommend the use of FFP for HA patients with these risk factors and stress the need to use alternative treatments to minimize complications and improve patient outcomes.

The following risk factor categories for treated patients include as few as 2 patients. The findings should therefore be interpreted with caution and not considered as confirmatory. HA patients may experience SLE due to immune system dysregulation or chronic inflammation associated with HA.^74^ HA can also be linked to SLE because of genetic predispositions or shared pathways involving complement system abnormalities.^75^ During an HA attack, increased vascular permeability could lead to neurological symptoms like seizures.^76–78^ We find lower mean LOS associated with SLE, seizure, and diabetes patients administered FFP. While there is little empirical evidence on the effects of FFP on SLE/seizure HA patients, some guidance exists regarding its viability for such cases.^79^ HA patients have similar rates of diabetes as the general population, and FFP infusion might not create complications for patients with only diabetes.^80^

We found that FFP used for older patients or those experiencing severe HA attacks was associated with higher mean LOS. Advanced age is often associated with physiological decline, making older HA patients more susceptible to severe and frequent attacks.^81^ Aging is linked to an increased burden of comorbidities and impaired immune function, which can complicate the treatment and management of HA.^82^ We also found that FFP usage was associated with lower mean LOS in moderate CCI scores; it was not used for patients with higher scores. These factors all indicate that FFP can cause complications for patients who are already vulnerable to complications.^83^ These findings highlight the need for careful consideration when using FFP in older HA patients or those with multiple comorbidities, as its potential risks may outweigh its benefits in this vulnerable subpopulation.

Regional disparities in healthcare access and treatment availability can significantly impact patient outcomes, particularly for those with rare diseases like HA. We find substantially higher mean LOS associated with patients treated in the Midwest region. FFP infusion can cause allergic reactions and complications, and with the lack of specialized care for rare diseases or monitoring available in the Midwest, it might not be suitable for administration in this region.^84,85^ The hospitals in this region might be in rural areas with an inability to access C1-INH therapies, which points to the need for the provision of these therapies for hospitals in these areas.^86–88^ We also find higher mean LOS for low-income and Medicare patients. This suggests the lack of access for these patients to newer therapies, delays in diagnosis/treatment, higher comorbidities making for challenging patient cases, and limited access to specialized care.^89,90^ Addressing these regional and socioeconomic disparities in healthcare access is crucial to improving patient outcomes, reducing hospital stays, and ensuring that all HA patients receive timely and effective treatment, particularly in underserved areas.

Gender differences in healthcare outcomes are important to consider, particularly when evaluating hospital LOS for HA patients. Male sex was associated with a higher mean LOS as than female sex. Males are likely to have higher comorbidities, greater disease severity, delays in getting medical treatment, and higher exposure to stress or triggers, factors that could be associated with longer hospital stays.^91–93^ Due to male physiology, FFP should be avoided for them to not aggravate their conditions. Male HA patients should make safer lifestyle choices and seek routine medical checkups to ensure early monitoring of comorbidities and requisite treatment. By recognizing the gender-related physiological differences in comorbidities and severity of disease, we can help improve the health and well-being of male HA patients, ultimately reducing hospital stays and enhancing quality of life.

## Limitations

The NIS data are based on insurance claims and may have medical coding issues and missing diagnoses. However, the accuracy of data collection for medical reimbursement is well-enforced in the United States. At-will observational databases such as NIS lack clinical data (lab results, imaging reports) and contain data from a sample of US hospitals, but not all hospitals participate. Nevertheless, the NIS^31–33^ is a comprehensive data source that enables researchers to conduct in-depth healthcare analysis.

It is important to acknowledge that the ICD-10 code D84.1 might not capture the entirety of HA patients; hence, the results need to be interpreted cautiously especially when generalizing to the HA population.

Due to the rarity of HA and the infrequent use of FFP infusion as a treatment modality, the number of patients receiving FFP in this study is inherently small. Certain risk factor categories for treated patients (eg, diabetes and renal conditions) include as few as 2 patients. This limits the statistical power to detect differences and increases uncertainty around estimates. The findings should therefore be interpreted with caution and not considered as confirmatory.

Patients with quicker discharges from the hospital could also experience adverse events such as rehospitalization. The question of hospitalization is out of scope for this research, as this study focuses on the LOS outcome for patients treated with FFP. Rehospitalization could be an avenue for further research. We acknowledge that alternative variable operationalizations (eg, different groupings of comorbidities, thresholds for prolonged LOS) could influence the observed associations. While our primary models were based on clinically grounded definitions, future work could explore the sensitivity of these findings to alternate operationalizing strategies. We also acknowledge that this is an observational study and not an experimental design, hence we have been careful to interpret the results as associations due to the presence of confounders. The results should be carefully interpreted and generalized.

## CONCLUSIONS

This analysis highlights the role of FFP in the treatment of HA patients, particularly in emergency settings. While FFP is occasionally used as a first-line intervention for acute attacks, especially when patients present through the ER, its administration was associated with longer hospital stays for patients with respiratory, cardiovascular disease, urticaria or those experiencing severe disease. These findings suggest that FFP may exacerbate underlying conditions, increase complication risk, and delay recovery in vulnerable populations.

### Disclosures

The authors declare no conflicts of interest and received no external funding for this research. All analyses and writing were completed independently without additional assistance.

## Supplementary Material

Online Supplementary Material

## Data Availability

To support reproducibility and transparency, author codes are available on request. All variables were taken from the NIS database as-is. The NIS 2021 database can be used to apply the codes and replicate results; however, patient-level data cannot be shared due to privacy legislation.

## References

[1] Castaldo A. J., Wells K. E., Khalid S.. (2025). Establishing a hereditary angioedema prevalence for the United States using a large administrative claims database. Ann Allergy Asthma Immunol.

[2] Frank M. M. (2006). Hereditary angioedema: The clinical syndrome and its management in the United States. Immunol Allergy Clin North Am.

[3] Cicardi M., Aberer W., Banerji A.. (2014). Classification, diagnosis, and approach to treatment for angioedema: consensus report from the Hereditary Angioedema International Working Group. Allergy.

[4] Cicardi M., Zuraw B. L. (2010). Hereditary angioedema: pathophysiology, diagnosis, and treatment. Immunol Allergy Clin North Am.

[5] Maurer M., Magerl M., Ansotegui I.. (2018). The international WAO/EAACI guideline for the management of hereditary angioedema—the 2017 revision and update. World Allergy Organ J.

[6] Bernstein J. A., Cremonesi P., Hoffmann T. K., Hollingsworth J. (2013). Disease severity and diagnostic delay in patients with hereditary angioedema: findings from the hereditary angioedema burden of illness study (HAE-BOIS-Europe). BMJ Open.

[7] Craig T., Aygören-Pürsün E., Bork K.. (2011). C1 inhibitor concentrate for acute treatment of hereditary angioedema attacks: a European registry. Allergy Asthma Proc.

[8] Zuraw B. L. (2018). Hereditary angioedema with normal C1 inhibitor: diagnosis and management. J Allergy Clin Immunol Pract.

[9] Cicardi M., Bergamaschini L. (2010). Hereditary angioedema: new treatment options. Drugs Today (Barc).

[10] Gerasimos C. (2018). Treatment patterns and outcomes of hereditary angioedema in North America. Clin Rev Allergy Immunol.

[11] Dang T. (2023). Real-world evidence in hereditary angioedema: outcomes with new therapies. Ther Adv Drug Saf.

[12] Zuraw B. L. (2017). Novel therapies for hereditary angioedema. Immunol Allergy Clin North Am.

[13] Halvorson S. A., Wang H. E., Kline J. A. (2021). Impact of hospital length of stay on patient outcomes and readmission rates. Am J Emerg Med.

[14] Smith R.K., Patel S.H., Lee T.C. (2020). Effectiveness of early intervention in reducing length of hospital stay for critically ill patients: a meta-analysis. J Intensive Care Med.

[15] Williams M. J., Patel N., Thomas S. (2022). Reducing hospital length of stay through quality improvement initiatives. J Hosp Med.

[16] Jones L. P., Taylor M., Williams K. (2021). Geographic disparities and hospital protocol differences in treatment-related length of stay. Health Serv Res.

[17] Craig T. J., Bernstein J. A., Farkas H., Bouillet L., Boccon-Gibod I. (2014). Diagnosis and treatment of bradykinin-mediated angioedema: outcomes from an angioedema expert consensus meeting. Int Arch Allergy Immunol.

[18] Morgan P. B., Price V. E., Lau K. K. (2020). Transfusion-associated circulatory overload: an under-recognized and potentially fatal complication. Transfus Med Rev.

[19] Pandey S., Vyas G. N. (2012). Adverse effects of plasma transfusion. Transfusion.

[20] Riedl M. A. (2015). Hereditary angioedema with normal C1-INH (HAE-nl-C1INH). J Allergy Clin Immunol Pract.

[21] Zuraw B. L., Christiansen S. C. (2013). HAE pathophysiology and underlying mechanisms. Allergy Asthma Proc.

[22] US Hereditary Angioedema Association (HAEA) Medical Advisory Board (2021). Guidelines for the management of hereditary angioedema.

[23] Prematta M., Gibbs J. G., Pratt E. L., Stoughton T. R., Craig T. J. (2007). Fresh frozen plasma for the treatment of hereditary angioedema. Ann Allergy Asthma Immunol.

[24] Banerji A., Riedl M. A., Castaldo A. J. (2018). Hospitalization rates and clinical outcomes in patients with hereditary angioedema. J Allergy Clin Immunol.

[25] Juffermans N. P., van der Poll T., Schultz M. J. (2015). Transfusion of fresh-frozen plasma in critically ill patients with coagulopathy: a systematic review. J Clin Anesth.

[26] Mayo Clinic (2023). Fresh Frozen Plasma transfusion in critically ill patients: a review of risks and benefits. Mayo Clin Proc.

[27] Yilmaz S., Gok M., Ozdemir L. (2018). Does transfusion of blood and blood products increase the length of stay in hospital?. Asian Cardiovasc Thorac Ann.

[28] Yilmaz S., Gok M., Ozdemir L. (2018). Does transfusion of blood and blood products increase the length of stay in hospital?. Asian Cardiovasc Thorac Ann.

[29] Barton C. W., Lane B. L., Gregory D. M. (2020). Impact of FFP in massive transfusion protocols on hospital length of stay and patient outcomes. Transfus Med Rev.

[30] Jain S., Sharma A., Kiran S. (2021). Effect of Fresh Frozen Plasma transfusion on length of stay and patient outcomes in critically ill patients. J Crit Care Med.

[31] Healthcare Cost and Utilization Project (HCUP) (2023). Overview of the National (Nationwide) Inpatient Sample (NIS).

[32] Elixhauser A., Steiner C., Harris D.R. (1998). Comorbidity measures for use with administrative data. Med Care.

[33] Zhan C., Miller M. R. (2003). Administrative data-based patient safety research: a critical review. Qual Saf Health Care.

[34] Kahn C. E., Jr., McDonald C. J. (2015). Big data in healthcare: challenges and opportunities. J Am Med Inform Assoc.

[35] Centers for Medicare & Medicaid Services (2023). ICD-10-CM Code D84.1: Defects in the Complement System.

[36] Charlson M. E., Pompei P., Ales K. L., MacKenzie C. R. (1987). A new method of classifying prognostic comorbidity in longitudinal studies: development and validation. J Chronic Dis.

[37] Quan H., Sundararajan V., Halfon P.. (2005). Coding algorithms for defining comorbidities in ICD-9-CM and ICD-10 administrative data. Med Care.

[38] Petri M. (2005). Review of classification criteria for systemic lupus erythematosus. Rheum Dis Clin North Am.

[39] Kaplan A.P. (2004). Chronic urticaria: pathogenesis and treatment. J Allergy Clin Immunol.

[40] Chipman H. A., George E. I., McCulloch R. E. (2010). BART: Bayesian additive regression trees. Ann Appl Stat.

[41] Linero A. R. (2018). Bayesian regression trees for high-dimensional prediction and variable selection. J Am Stat Assoc.

[42] Pratola M. T. (2016). Efficient Metropolis-Hastings proposal mechanisms for Bayesian regression tree models. Bayesian Anal.

[43] Denison D. G., Mallick B. K., Smith A. F. M. (1998). Bayesian MARS. Stat Comput.

[44] Murray J. S. (2017). Log-linear Bayesian additive regression trees for categorical and count responses.

[45] Hill J. L. (2011). Bayesian nonparametric modeling for causal inference. J Comput Graph Stat.

[46] Sparapani R. A., Spanbauer C., McCulloch R. E. (2021). Nonparametric machine learning and efficient computation with Bayesian additive regression trees: the BART R package. J Stat Softw.

[47] Kay M., Mastny T. (2023). Tidybayes: Tidy Data and Geoms for Bayesian Models.

[48] Austin P. C., Tu J. V. (2004). Automated variable selection methods for logistic regression produced unstable models for predicting acute myocardial infarction mortality. J Clin Epidemiol.

[49] Goldstein H. (2011). Multilevel Statistical Models.

[50] Chipman H. A., George E. I., McCulloch R. E. (2010). BART: Bayesian additive regression trees. Ann Appl Stat.

[51] Linero A. R. (2018). Bayesian regression trees for high-dimensional prediction and variable selection. J Am Stat Assoc.

[52] Kapelner A., Bleich J. (2015). Prediction with missing data via Bayesian additive regression trees. Can J Stat.

[53] Pratola M. T. (2016). Efficient Metropolis-Hastings proposal mechanisms for Bayesian regression tree models. Bayesian Anal.

[54] Tan Y., Roy J., Yoon J. (2021). Scalable Bayesian modeling using BART in high-dimensional settings. Stat Med.

[55] Gelman A., Carlin J. B., Stern H. S., Dunson D. B., Vehtari A., Rubin D. B. (2013). Bayesian Data Analysis.

[56] Zuraw B. L. (2008). Hereditary angioedema. N Engl J Med.

[57] Bernstein J. A., Cremonesi P., Hoffmann T. K., Hollingsworth J. (2017). Current treatment of hereditary angioedema: an update on the approved and investigational drugs. Exp Opin Investig Drugs.

[58] Cicardi M., Banerji A., Bracho F.. (2010). Icatibant, a new bradykinin-receptor antagonist, in hereditary angioedema. N Engl J Med.

[59] Craig T., Aygören-Pürsün E., Bork K.. (2012). WAO guideline for the management of hereditary angioedema. World Allergy Organ J.

[60] Bork K., Meng G., Staubach P., Hardt J. (2006). Hereditary angioedema: new findings concerning symptoms, affected organs, and course. Am J Med.

[61] Caballero T., Canabal J., Prior N.. (2018). Management of airway in hereditary angioedema: a challenge for emergency physicians. J Inv Allergol Clin Immunol.

[62] Prematta M., Gibbs J. G., Pratt E. L., Stoughton T. R., Craig T. J. (2007). Fresh frozen plasma for the treatment of hereditary angioedema. Ann Allergy Asthma Immunol.

[63] Nzeako U. C., Longhurst H. J. (2019). Hereditary angioedema: current treatment options and decision making. Allergy Asthma Clin Immunol.

[64] Longhurst H., Cicardi M. (2012). Hereditary angio-oedema. Lancet.

[65] Lumry W. R. (2018). Hereditary angioedema: the economics of treatment of an orphan disease. Front Med (Lausanne).

[66] Zuraw B. L., Christiansen S. C. (2020). HAE therapies: past, present, and future. Allergy Asthma Proc.

[67] Reshef A., Prematta M. J., Craig T. J. (2013). Stress and hereditary angioedema: a survey of patients. Allergy Asthma Proc.

[68] Riedl M. A. (2013). Urticaria and angioedema: a practical approach. Allergy Asthma Proc.

[69] Poonawalla T., Kelly B. (2009). Urticaria: a review. Am J Clin Dermatol.

[70] Kaplan A. P., Adkinson N. F., Jr. (2014). Middleton’s Allergy: Principles and Practice.

[71] Zuraw B. L. (2008). Hereditary angioedema. N Engl J Med.

[72] Sampson H. A. (2017). Anaphylaxis: past, present, and future. Allergy Asthma Proc.

[73] Patel K. V., Pandey A., Kapoor A.. (2021). Hereditary angioedema and cardiovascular complications: a review. Am J Cardiovasc Drugs.

[74] Williamson L. M., Allain J. P. (1995). Virally inactivated fresh frozen plasma. Vox Sang.

[75] Doria A., Zen M., Canova M.. (2010). SLE diagnosis and treatment: when early is early. Autoimmun Rev.

[76] Enocsson H., Wetterö J., Sjöwall C. (2021). Interferon-ɑ and immune activation in systemic lupus erythematosus. Front Immunol.

[77] Machado C., Araújo M., Pinto J.. (2022). Seizures in hereditary angioedema: a rare but serious manifestation. Epilepsy Behav Rep.

[78] Longhurst H. J., Bork K. (2006). Hereditary angioedema: causes, manifestations and treatment. Br J Hosp Med.

[79] Farkas H. (2013). Current pharmacotherapy of bradykinin-mediated angioedema. Expert Opin Pharmacother.

[80] Abolhassani H., Sagvand B. T., Shokuhfar T.. (2015). Treatment strategies for angioedema in patients with immune dysregulation. J Clin Immunol.

[81] Banerji A., Bernstein J. A., Wang Y.. (2020). Effectiveness of hereditary angioedema treatments in patients with coexisting medical conditions. Ann Allergy Asthma Immunol.

[82] Cugno M., Zanichelli A., Foieni F., Caccia S., Cicardi M. (2009). C1-inhibitor deficiency and angioedema: molecular mechanisms and clinical progress. Trends Mol Med.

[83] Bernard S., Luu M., Dai D.. (2021). The impact of age on the prevalence of comorbid conditions in hereditary angioedema. J Allergy Clin Immunol Pract.

[84] Jackson C. H., Sharples L. D., Thompson S. G. (2002). Structural and predictive models for health outcomes in the elderly. Stat Methods Med Res.

[85] Roberts I., Ramesh R. (2021). Access to care for rare diseases in rural America: the case for targeted policy intervention. J Rural Health.

[86] Miesfeldt S., Murray K., Lucas L., Chang C. H., Li Z. (2017). Closing the rural mortality gap: improving emergency care for rare conditions. JAMA.

[87] Hennessy D. A., Kaplan G. G., Maxwell C. J.. (2020). Access to therapies for rare diseases in rural vs urban areas: a population-based study. Orphanet J Rare Dis.

[88] Thomas S. D., Smith K. J., Klabunde C. N. (2018). Disparities in access to new therapies for rare diseases: rural and urban perspectives. Med Care.

[89] Gamm L., Hutchison L., Dabney B. (2003). Rural healthy people 2010: a companion document to Healthy People 2010.

[90] Goldman D. P., Joyce G. F., Zheng Y. (2007). Prescription drug cost sharing: associations with medication and medical utilization and spending and health. JAMA.

[91] Veenstra D. L., Gibson T. B., Faries D. E.. (2018). Socioeconomic status and outcomes for patients with rare diseases. Pharmacoeconomics.

[92] Madsen F., Atar D., Brorsson B.. (2019). Gender differences in hospital resource use and outcome in patients with angioedema: a multinational cohort study. BMC Emerg Med.

[93] Kwon Y. S., Park S. H., Kim T. H.. (2018). Gender differences in the prevalence and comorbidity of angioedema. Clin Exp Allergy.

