# Supplementary material for: Effect of Fresh Frozen Plasma Infusion on Hospital Length of Stay for Patients With Hereditary Angioedema
Source: J Health Econ Outcomes Res. 2025 Jul 9;12(2):1–10. doi: 10.36469/001c.141171 (PMC12251561; doi:10.36469/001c.141171)
Supplement: Online Supplementary Material [file jheor_2025_12_2_141171_293432.pdf]

Journal of Health Economics and Outcomes Research

Online Supplementary Material

Effect of Fresh Frozen Plasma Infusion on Hospital Length of Stay for Patients With Hereditary Angioedema. *JHEOR*. 2025;12(2):1-10. doi:10.36469/jheor.2025.141471

Table S1: Patient Demographics in the Hereditary Angioedema Cohort

Table S2: Comorbidities and Risk Factors During Hospitalization for the Hereditary Angioedema Cohort

Table S3: Emergency Room Admission and FFP Treatment for the Hereditary Angioedema Cohort

Table S4: ICD-10 Diagnosis Codes for Patient Risk Factors

Figure S1: Average Inclusion of Variables in the (A) BART Model for Each MCMC Iteration and (B) the FFP Treatment Variable in the BART Model for MCMC Iteration Where Treatment Was Included

This supplementary material has been provided by the authors to give readers additional information about their work.

This is an open-access article distributed under the terms of the Creative Commons Attribution 4.0 International License (CCBY-4.0). View this license's legal deed at http://creativecommons.org/licenses/by/4.0 and legal code at http://creativecommons.org/licenses/by/4.0/legalcode for more information.

Table S1. Patient Demographics in the Hereditary Angioedema Cohort

	**Overall (N=441)**		**Treatment (N=29)**	
	**n**	**%**	**n**	**%**
Age (y)				
0 -17	37	8.4	1	0.2
18-39	146	33.1	13	2.9
40-64	170	38.5	10	2.3
≥65	88	20.0	5	1.1
Sex				
Female	310	70.3	21	72.4
Male	131	29.7	8	27.6
Race				
White	258	58.5	16	55.2
Black	89	20.2	3	10.3
Hispanic	58	13.2	4	13.8
Other[a]	36	8.2	6	20.7
Income quartile				
First	109	24.7	5	17.2
Second	110	24.9	11	37.9
Third	125	28.3	10	34.5
Fourth	93	21.1	3	10.3
Pay type				
Private	172	39.00	6	20.7
Medicare	127	28.80	7	24.1
Medicaid	112	25.40	12	41.4
Other[b]	30	6.80	4	13.8
Region				
Northeast	90	20.4	8	27.6
Midwest	87	19.7	3	10.3
South	162	36.7	8	27.6
West	102	23.1	10	34.5

[a]Includes Asian, Native American, other.
[b]Includes self-pay, other sources.

Table S2. Comorbidities and Risk Factors During Hospitalization for the Hereditary Angioedema Cohort

	**Overall (N=441)**		**Treatment (N=29)**	
	**n**	**%**	**n**	**%**
Comorbidities				
Hypertension	212	48.1	10	34.5
Obesity	114	25.9	9	31.0
Chronic obstructive pulmonary disease	107	24.3	8	27.6
Diabetes	92	20.9	4	13.8
Depression	71	16.1	6	20.7
Thyroid	65	14.7	1	3.4
Autoimmune disease	64	14.5	1	3.4
Drug abuse	28	6.3	2	6.9
Peripheral vascular disease	20	4.5	0	0.0
Alcohol abuse	13	2.9	4	13.8
Charlson Comorbidity Index score				
0	90	20.4	7	24.1
1	83	18.8	6	20.7
2	90	20.4	7	24.1
3	87	19.7	7	24.1
4	56	12.7	1	3.4
5	27	6.1	0	0.0
6	5	1.1	1	3.4
7	2	0.5	0	0.0
8	1	0.2	0	0.0
Risk factors				
Respiratory	97	22.0	11	37.9
Diabetes	85	19.3	2	6.9
Cardiovascular disease	60	13.6	4	13.8
Systemic lupus erythematosus	57	12.9	1	3.4
Renal disease	51	11.6	2	6.9
Urticaria	50	11.3	5	17.2
Chronic obstructive airway disease	36	8.2	0	0.0
Seizures	26	5.9	2	6.9
Sepsis	17	3.9	2	6.9
Emergency room admission				
Yes	334	75.7	28	96.6
No	107	24.3	1	3.4

Table S3. Emergency Room Admission and FFP Treatment for the Hereditary Angioedema Cohort (N=441)

	n	%
FFP treated		
Yes	29	6.6
No	412	93.4
No. of FFP Infusions		
1	25	5.7
2	4	0.9
Days to first FFP infusion		
0	19	4.3
1	9	2.0
2	1	0.2

Table S4. ICD-10 Diagnosis Codes for Patient Risk Factors

Respiratory	J96*, J39*, J81*, J95*
Diabetes	E11*
Cardiovascular disease	I11*, I50*
Systemic lupus erythematosus	M32*
Renal disease	N170*, N186*, I13*
Urticaria	T78*
Chronic obstructive airway disease	J44*
Seizures	G40*
Sepsis	R652*

Figure S1. Average Inclusion of Variables in the (**A**) BART Model for Each MCMC Iteration and (**B**) the FFP Treatment Variable in the BART Model for MCMC Iteration Where Treatment Was Included

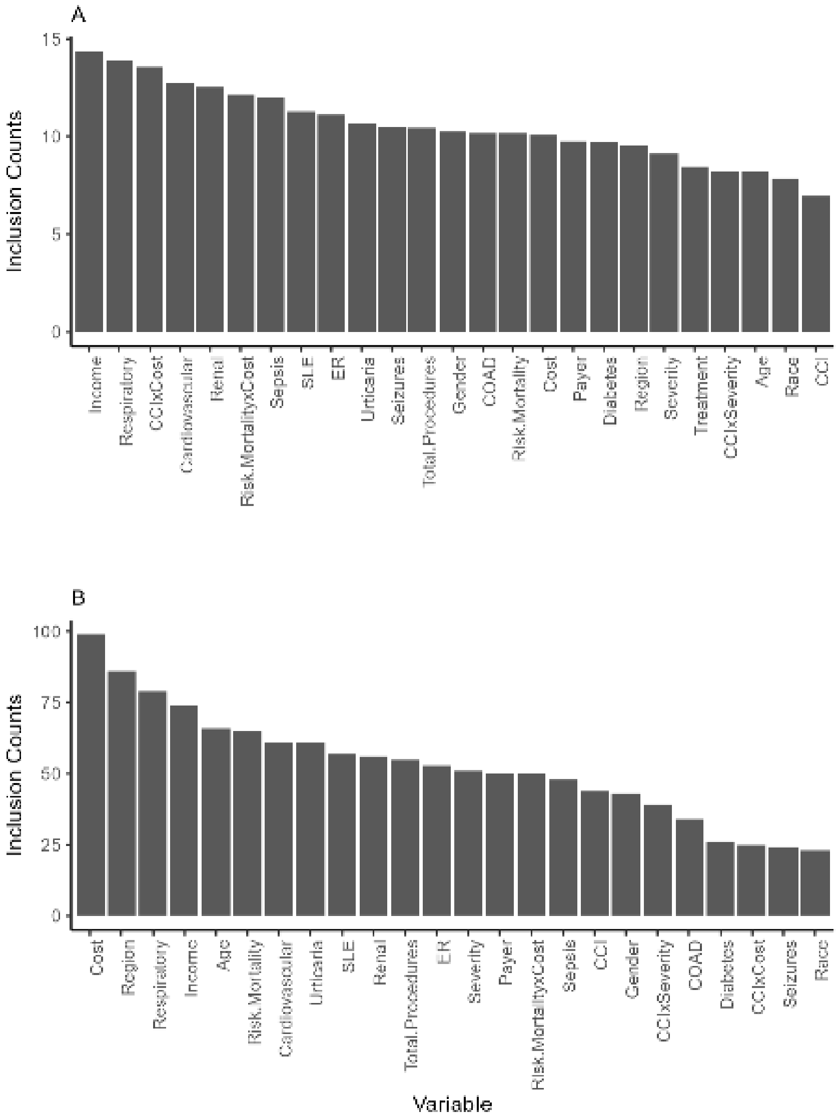

Abbreviations: CCI, Charlson Comorbidity Index; COAD, chronic obstructive airway disease; ICD-10, *International Classification of Diseases, Tenth Revision*; SLE, systemic lupus erythematosus.
